# Intra-Arterial TheraSphere Yttrium-90 Glass Microspheres in the Treatment of Patients With Unresectable Hepatocellular Carcinoma: Protocol for the STOP-HCC Phase 3 Randomized Controlled Trial

**DOI:** 10.2196/11234

**Published:** 2018-08-15

**Authors:** Nikhil Chauhan, Janet Bukovcan, Eveline Boucher, David Cosgrove, Julien Edeline, Bonnie Hamilton, Laura Kulik, Fayaz Master, Riad Salem

**Affiliations:** ^1^ Research and Development BTG International Group Companies London United Kingdom; ^2^ Division of Medical Oncology Compass Oncology Vancouver Cancer Center Vancouver, WA United States; ^3^ Department of Oncology Centre Eugene Marquis Rennes France; ^4^ Division of Gastroenterology and Hepatology Department of Medicine Northwestern Memorial Hospital Chicago, IL United States; ^5^ Section of Interventional Radiology Department of Radiology Northwestern University Chicago, IL United States; ^6^ Division of Hematology and Oncology Department of Medicine Northwestern University Chicago, IL United States; ^7^ Division of Transplant Surgery Department of Surgery Northwestern University Chicago, IL United States

**Keywords:** sorafenib, hepatocellular carcinoma, microspheres, yttrium radioisotopes, research design, clinical trial, phase III, randomized controlled trial, carcinoma, hepatocellular

## Abstract

**Background:**

Globally, hepatocellular carcinoma is the second most common cause of cancer deaths. It remains challenging to intensify cancer treatment without impairing liver function.

**Objective:**

The objective of the TheraSphere in the Treatment of Patients with Unresectable Hepatocellular Carcinoma (STOP-HCC) study is to examine the hypothesis that transarterial radioembolization (TheraSphere yttrium-90 glass microspheres) combined with standard first-line treatment with sorafenib will improve outcomes over treatment with sorafenib alone in unresectable hepatocellular carcinoma. The STOP-HCC study is the largest international, multicenter, prospective study of intra-arterial treatment in combination with sorafenib in unresectable hepatocellular carcinoma. Here we report the study design.

**Methods:**

STOP-HCC is a prospective, phase 3, open-label, randomized controlled study conducted across up to 105 sites in North America, Europe, and Asia. Eligible adults have unresectable hepatocellular carcinoma and a life expectancy of at least 12 weeks, 1 or more unidimensional measurable lesions, Child-Pugh score 7 points or less, and Eastern Cooperative Oncology Group Performance Status score 1 or lower, and are candidates for treatment with sorafenib. Presence of branch portal vein tumor thrombosis is permitted. Patients were randomly assigned in a 1:1 ratio to receive either sorafenib alone or transarterial radioembolization followed by sorafenib within 2 to 6 weeks. The primary outcome is overall survival. Secondary outcomes are time to progression, time to untreatable progression, time to symptomatic progression, tumor response, quality of life, and adverse event occurrence. The study is an adaptive trial, comprising a group-sequential design with 2 interim analyses with 520 patients, and an option to increase the sample size to 700 patients at the second interim analysis. The sample size of 520 patients allows for 417 deaths to give 80% power to detect an increase in median overall survival from 10.7 months for the sorafenib group (based on the Sorafenib Hepatocellular Carcinoma Assessment Randomized Protocol [SHARP] trial) to 14.2 months for the transarterial radioembolization+sorafenib group (hazard ratio 0.754) with 2-sided alpha of .05. The increased sample size of 700 patients allows for 564 deaths to give 80% power to detect a smaller difference in median overall survival from 10.7 months for the sorafenib group to 13.7 months for the transarterial radioembolization+sorafenib group (hazard ratio 0.781).

**Results:**

Enrollment for the study completed in September 2017. Results of the first and second interim analyses were reviewed by the Independent Data Monitoring Committee. The recommendation of the committee, at both interim analyses, was to continue the study without any changes.

**Conclusions:**

The STOP-HCC study will contribute toward the establishment of the role of combination therapy with transarterial radioembolization and sorafenib in the treatment of unresectable hepatocellular carcinoma with and without branch portal vein tumor thrombosis.

**Trial Registration:**

ClinicalTrials.gov NCT01556490; https://clinicaltrials.gov/ct2/show/NCT01556490 (Archived by WebCite at http://www.webcitation.org/7188iygKs).

**Registered Report Identifier:**

RR1-10.2196/11234

## Introduction

### Background

Primary liver cancer (hepatocellular carcinoma [HCC] and intrahepatic bile duct cancer) is the fifth most common cancer in men and ninth most common in women worldwide [1]. Globally, HCC is the second most common cause of cancer deaths [1]. The selection of therapeutic strategy and prediction of survival are guided by disease staging systems, of which the most widely used is the Barcelona Clinic Liver Cancer (BCLC) classification system [2-4]. The system determines cancer stage (BCLC A, B, C, and D) and prognosis based on 3 major prognostic factors of HCC: tumor burden, liver function, and Eastern Cooperative Oncology Group (ECOG) Performance Status score. This allows differentiation of patients with early-stage disease, who could benefit from curative treatments, from those with intermediate, advanced, and end-stage disease, who mostly receive palliative therapies or best supportive care.

Early-stage disease is often asymptomatic and unlikely to be diagnosed unless patients are enrolled in a surveillance program. Most HCC is diagnosed after patients have progressed beyond early-stage disease and, thus, they receive palliative treatments (intra-arterial treatment, systemic therapies, and external radiation), with the goal to improve life expectancies and maintain a good of quality of life. Median survival in this group of patients varies widely, ranging from approximately 3 months for patients with metastatic disease and portal vein thrombosis to up to 33 months for patients with good prognosis [5,6]. The patients with a diagnosis of BCLC D disease are not expected to derive a significant therapeutic benefit from HCC treatment unless they are bridged to transplantation. Even among those for whom treatment remains possible, most patients receive only best supportive care [7].

Patients with unresectable HCC are primarily treated with locoregional therapies and systemic agents. The systemic treatment sorafenib (Nexavar, Bayer HealthCare Pharmaceuticals Inc, Wayne, New Jersey, USA) is the only approved systemic therapy in the first-line treatment of patients with unresectable HCC [8,9]. It is the standard-of-care treatment for patients with advanced HCC (BCLC C), as recommended by the European Association for the Study of the Liver [3]; however, some regional differences exist for patient selection for sorafenib treatment [10]. Two placebo controlled, randomized, phase 3 trials (Sorafenib Hepatocellular Carcinoma Assessment Randomized Protocol [SHARP] and the Asia-Pacific trial) demonstrated that sorafenib provided a significant improvement in median overall survival [11,12]. In the SHARP trial, median overall survival improved from 7.9 months in the placebo group to 10.7 months in the sorafenib group (hazard ratio [HR] 0.69, 95% CI 0.55-0.87) and, in the Asia-Pacific trial, median overall survival improved from 4.2 months in the placebo group to 6.5 months in the sorafenib group (HR 0.68, 95% CI 0.50-0.93). Individual responses to sorafenib may be affected by baseline characteristics, such as disease etiology, tumor burden, performance status, tumor stage, and prior therapy. Sorafenib consistently increased median overall survival across these different subgroups, compared with placebo, as reflected by HRs of 0.50 to 0.85, which were similar to that of the overall group (HR 0.69) [13]. The subgroups with HRs in the lower end of this range were patients with an ECOG Performance Status score of 1 to 2 (median overall survival 8.9 months with sorafenib vs 5.6 months with placebo; HR 0.71, 95% CI 0.52-0.96); patients with both extrahepatic disease and portal vein invasion (median overall survival 8.9 months with sorafenib vs 6.7 months with placebo; HR 0.77, 95% CI 0.60-0.99); and BCLC C patients (median overall survival 9.7 months with sorafenib vs 7.0 months with placebo; HR 0.70, 95% CI 0.56-0.89) [13].

The use of sorafenib in clinical practice was assessed in over 3000 patients in the global observational registry study of sorafenib, Global Investigation of Therapeutic Decisions in Hepatocellular Carcinoma and of its Treatment With Sorafenib (GIDEON) [14]. Median overall survival in GIDEON ranged from 8.5 months (95% CI 7.6-9.6) in the United States to 14.5 months (95% CI 13.2-17.4) in Japan [10]. Despite the indisputable benefit of sorafenib, 85% of patients experienced at least one adverse event, and the incidence of drug-related adverse events leading to discontinuation was around 31% in the GIDEON registry [10].

After the marketing of sorafenib was approved in North America, numerous studies that evaluated other molecular therapies did not demonstrate their superiority or noninferiority to sorafenib [15,16]. The combination of locoregional therapy with transarterial chemoembolization (TACE) and targeted agents (sorafenib, brivanib, and orantinib) initially appeared to be an attractive strategy; however, the combinations did not improve overall survival in 5 large randomized trials [17]. Recent significant advancements were the approval of regorafenib (Stivarga, Bayer HealthCare Pharmaceuticals Inc, Whippany, NJ, USA) and nivolumab (Opdivo, Bristol-Myers Squibb Co., Princeton, NJ, USA) for patients who have been previously treated with sorafenib [18-21]. Additional recent developments were phase 3 trials that demonstrated superiority of cabozantinib over placebo in the second-line treatment of advanced HCC and noninferiority of lenvatinib to sorafenib in the first-line treatment of unresectable HCC, as well as a phase 2 trial in which pembrolizumab showed encouraging results [22-24].

Transarterial radioembolization (TARE) could be an alternative to TACE treatment [25]. The 2 available TARE devices are TheraSphere microspheres (Biocompatibles UK Ltd, Surrey, UK) and SIR-Spheres resin microspheres (Sirtex Medical Ltd, North Sydney, Australia). Favorable survival outcomes with TheraSphere microspheres were reported for a large prospective cohort of 1000 patients over a 15-year period ending in March 2017 [26]. The majority (89%) of patients were treatment naïve. Median overall survival ranged between 8.0 and 47.3 months depending on disease stage and liver function. Also, TARE was well tolerated, and the adverse events rate was low compared with rates with systemic treatment or TACE: grade 3/4 albumin and bilirubin toxicities, respectively, were observed in 49 (5%) and 110 (11%) patients [26].

There is more rationale to combine sorafenib with TARE than with TACE. First, TARE is better tolerated than TACE, which may allow a higher dose and longer length of therapy with sorafenib. Additionally, sorafenib may be potentially more synergistic with yttrium-90, as antiangiogenic agents potentiate the effects of radiation on tumor regression [27].

Several small nonrandomized studies that investigated combination treatment with TARE and sorafenib reported favorable results among patients with BCLC A and B status. Median overall survival ranged between 12 and 20 months, and disease control was achieved in up to 100% of patients [27-30]. The most common toxicities reported were fatigue, diarrhea, and hand-foot syndrome. These toxicities are similar to those reported with sorafenib monotherapy. Sorafenib doses were reduced in up to 65% of patients and administration was interrupted in up to 13.8%.

Two large randomized multicenter trials were designed to evaluate combination treatment with TARE and sorafenib, with the primary end point of overall survival. The Evaluation of Sorafenib in Combination With Local Micro-Therapy Guided by Primovist Enhanced MRI in Patients With Hepatocellular Carcinoma (SORAMIC) trial compared sorafenib alone against TARE using resin yttrium-90 microspheres combined with sorafenib in patients with advanced HCC [31]. Recently reported results of the study, conducted primarily in Europe, showed that in a total of 419 patients, the combination of TARE and sorafenib did not improve overall survival over treatment with sorafenib alone (12.1 vs 11.5 months; HR 1.018, *P*=.87) and resulted in an increased rate of adverse events of grade 3 or higher (73% vs 65%) [32]; however, an overall survival benefit was observed in some subgroups.

### Objective

The TheraSphere in the Treatment of Patients with Unresectable Hepatocellular Carcinoma (STOP-HCC) study is the largest phase 3 prospective study of TARE in combination with sorafenib in unresectable advanced HCC. Compared with the SORAMIC trial, STOP-HCC has a larger sample size and so should have greater statistical power to detect a difference in overall survival; additionally, STOP-HCC uses glass yttrium-90 microspheres and is being conducted worldwide. At the time the STOP-HCC study was designed (in 2011), sorafenib was the only systemic agent approved for use as first-line treatment in HCC and, since then, there have been several trials reporting positive outcomes [10,33]. The primary purpose of the STOP-HCC study is to compare overall survival in patients who receive sorafenib alone with overall survival in patients who receive TARE with TheraSphere yttrium-90 microspheres followed by sorafenib. Here we report the protocol for the STOP-HCC study.

## Methods

### Overview

The STOP-HCC study, also known as the BTG TS-103 study, was registered with ClinicalTrials.gov (NCT01556490) on March 13, 2012. Textbox 1 describes eligibility criteria for the STOP-HCC study. Participating institutions obtained institutional review board approval of the protocol and informed consent form and obtained written informed consent from patients (Multimedia Appendix 1). Each institution has qualified investigators and support staff who conduct the study according to Good Clinical Practice guidelines, have adequate expertise in the treatment of patients with HCC, and are experienced with and trained in the use of TheraSphere yttrium-90 microspheres. A list of sites is available on the study’s ClinicalTrials.gov registration page (linked in the abstract).

**Patient eligibility for the TheraSphere in the Treatment of Patients with Unresectable Hepatocellular Carcinoma (STOP-HCC) study.**

**Inclusion criteria**

- Patients aged >18 years with unresectable hepatocellular carcinoma (HCC; confirmed by histology or noninvasive American Association for the Study of Liver Diseases criteria) who have a life expectancy ≥12 weeks.
- Measurable disease defined as at least one unidimensional measurable lesion by computed tomography or magnetic resonance imaging (according to Response Evaluation Criteria in Solid Tumors v1.1).
- Child-Pugh score ≤7 points and Eastern Cooperative Oncology Group Performance Status score of ≤1.
- Eligible to receive standard-of-care sorafenib.
- Platelet count >50×10^9^/L or >50% prothrombin activity; hemoglobin ≥8.5 g/dL (≥85.0 g/L); bilirubin ≤2.5 mg/dL (≤42.8 μmol/L); alanine aminotransferase and aspartate aminotransferase <5 times the upper limit of normal; amylase or lipase ≤2 times the upper limit of normal; serum creatinine ≤1.5 times the upper limit of normal; international normalized ratio for prothrombin time <2.0.

**Exclusion criteria**

*Key exclusions:*

- Eligible for curative treatment (eg, ablation or transplantation).
- Main portal vein thrombosis (branch portal vein thrombosis is permissible).
- Extrahepatic disease, except lung nodules and mesenteric or portal lymph nodes ≤2.0 cm each.

*Other exclusions:*

- Must not have tumor replacement >70% of total liver volume based on visual estimation by the investigator or must not have tumor replacement >50% of total liver volume in the presence of albumin <3 g/dL (<30 g/L).
- History of previous or concurrent cancer other than HCC unless treated curatively ≥5 years prior to entry.
- Contraindication to TheraSphere administration according to package label.
- Patients infected with HIV can be considered; however, they must be well managed and well controlled with an undetectable viral load.
- Contraindications to sorafenib, angiography, and selective visceral catheterization.
- Concurrent treatment with substrate agents for cytochrome P450 2B6, uridine diphosphate glucuronosyltransferase 1A1 or 1A9, and P-glycoprotein; rifampicin; St John’s wort; phenytoin; carbamazepine; phenobarbital; dexamethasone; other systemic anticancer agents (eg, docetaxel, doxorubicin, or irinotecan); other locoregional therapies (other than study treatment).
- Prior treatment with transarterial chemoembolization (TACE) or bland embolization must have occurred >2 months prior to randomization and must have been applied to a treatment field or lobe that is not to be treated under this protocol. For patients with tumor progression in the treatment field or lobe previously treated with TA(C)E, vessels feeding the tumor(s) must be assessed for adequate blood flow using angiography (cone beam computed tomography strongly recommended), and the TACE or bland embolization must have been applied >6 months prior to randomization.
- Prior receipt of external beam radiation treatment to the chest, liver, or abdomen.

### Overview of Study Design

STOP-HCC is an ongoing phase 3, randomized, parallel-group, multicenter, prospective, open-label study evaluating treatment with TheraSphere microspheres in patients with unresectable HCC in whom sorafenib therapy is already planned. Patients were recruited at up to 105 sites in the United States, Canada, Europe, and Asia. Eligible patients were randomly assigned in a 1:1 ratio to the sorafenib and TARE+sorafenib groups. The sorafenib group receives planned sorafenib according to the product label. The TARE+sorafenib group receives TheraSphere microspheres prior to the initiation of sorafenib. All patients are followed prospectively from randomization to death until the predefined number of deaths, to allow the final analysis to be conducted, have occurred.

Site investigators are from departments of radiology, nuclear medicine, and interventional radiology. Investigators are experienced in radioembolization with radioactive microsphere products. For sites with investigators with low or no experience with TheraSphere microspheres, before inclusion in the trial, the site team must have completed training that included 3 to 5 administrations of TheraSphere microspheres. Administration of TheraSphere microspheres is generally considered to be an outpatient procedure in the United States and Canada and an inpatient procedure in Europe and Asia. The physical location for aftercare and recovery was determined by individual institutional policies and facility configurations. The sites are collecting the data.

An Independent Data Monitoring Committee (IDMC), led by the IDMC chairperson, was established to oversee the conduct of the study. The IDMC met periodically to review enrollment, protocol deviations, and safety events. In addition, the IDMC conducted and reviewed an initial feasibility safety analysis and will evaluate the overall survival data at interim analyses for consideration of stopping the study for efficacy and for the option to increase the sample size at the second interim analysis. The IDMC is tasked to make formal recommendations to the study sponsor at the time of the feasibility safety analysis, at the time of the interim analyses, and during the conduct of the study based on detailed decision rules specified in the IDMC charter. An IDMC member or designate can act as the study independent medical monitor. The IDMC will evaluate the final study report.

### Screening

On day –14 to day 0, the following assessments were conducted: review of eligibility criteria, demographics, medical history, physical examination, medication and prior treatment history, serum pregnancy test for women, ECOG Performance Status, hematology (white blood cells, hemoglobin, hematocrit, and platelets), coagulation (prothrombin time, partial thromboplastin time, and international normalized ratio for prothrombin time), chemistry panel, liver function tests, tumor markers for HCC (alpha-fetoprotein), triple-phase magnetic resonance imaging and spiral computed tomography (CT) of abdomen, Child-Pugh score, spiral CT of chest and pelvis, quality-of-life questionnaire, assessment and report of adverse events, and review and recording of concurrent medications. Informed consent was obtained at screening.

### Randomization and Stratification

Eligible patients were randomly assigned on day 0 using a computer-generated randomization scheme, and investigators had the option to randomly assign patients using either a Web-based electronic system or telephone. To create a balance between the 2 study groups, patients were stratified based on region (North America and Europe vs Asia), ECOG Performance Status (0 vs 1), and presence or absence of branch portal vein tumor thrombosis (PVTT). Randomly assigned patients who were unable to receive the planned study treatment continued to be followed under their assigned study group and will be included in the statistical analysis.

### Treatment

**Figure 1:**
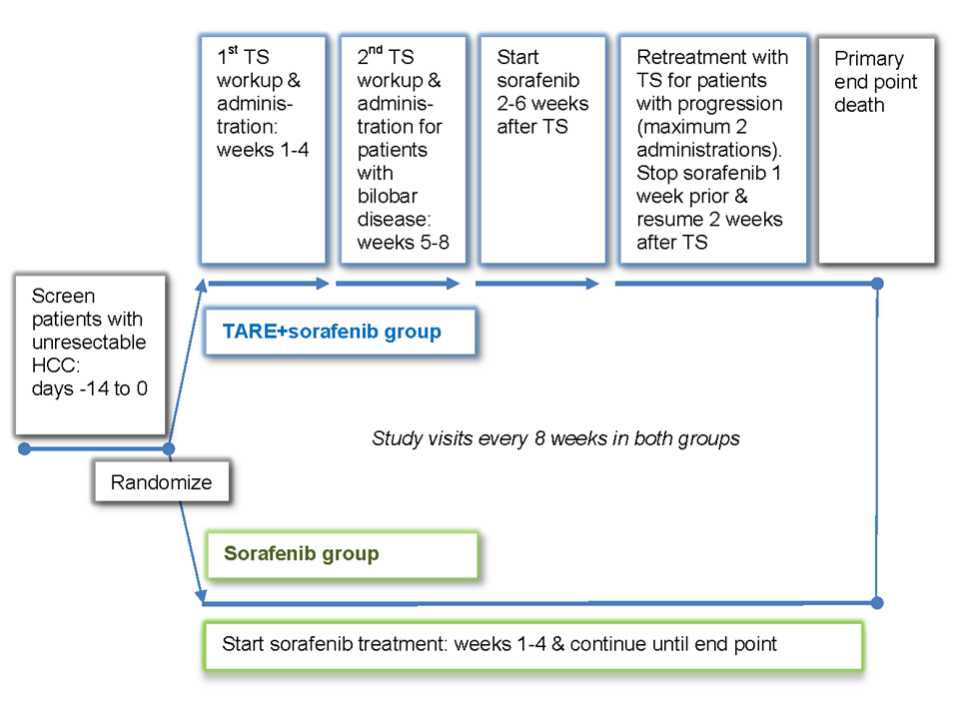
Clinical trial schema for the TheraSphere in the Treatment of Patients with Unresectable Hepatocellular Carcinoma (STOP-HCC) study. TARE: transarterial radioembolization; TS: TheraSphere microspheres.

Figure 1 shows the STOP-HCC treatment schema.

Table 1 shows the schedule of assessments and treatment. For patients who discontinued the study treatment due to progressive disease and were unable to maintain routine clinic visits, investigative sites should maintain telephone contact until death.

### Sorafenib Group

Patients randomly assigned to the sorafenib group start sorafenib as soon as possible after randomization, in accordance with the product labeling. Doses are adjusted over several visits as needed. Every 8 weeks after randomization, assessments are conducted as Table 1 describes.

**Table 1:** TheraSphere in the Treatment of Patients with Unresectable Hepatocellular Carcinoma (STOP-HCC) study schedule of assessments and events.

Interventions and Assessments		Sorafenib group: initiate treatment weeks 1-4; continue therapy weeks ≥5	TARE^a^+sorafenib group				Follow-up every 8 weeks ±14 days^c^ (for both groups)
			First workup and TARE treatment: Weeks 1-4	Second workup and TARE treatment for patients with bilobar disease: Weeks 5-8	Sorafenib treatment: initiate and continue sorafenib treatment >2 to <6 weeks after TARE	Workup and retreatment^b^ for patients with hepatic progression	
**Interventions**							
	Administer sorafenib^d^	✓			✓	✓^c^	
	Calculate liver volume and mass		✓			✓	
	Hepatic angiogram, ^99m^Tc-MAA scan^f^, calculate TARE dose, administer TARE^g^		✓	✓		✓	
**Assessments**							
	ECOG^h^ Performance Status		✓	✓		✓	✓^i^
	Hematology^j^, coagulation^k^, chemistry panel, liver function tests	✓^l^	✓^l^			✓	✓^m^
	Tumor markers for hepatocellular carcinoma (alpha-fetoprotein)	✓	✓				✓^m^
	Triple-phase MRI^n^/spiral CT^o^ abdomen, Child-Pugh score, spiral CT chest and pelvis, quality-of-life questionnaire						✓^m^
	Assess and report adverse events, review and record concurrent medication	✓	✓	✓		✓	✓^m^
	Final end point, efficacy and safety documentation, and exit patient						✓^i^

^a^TARE: transarterial radioembolization.

^b^Additional TARE workup and administration in lesions amenable to further TARE.

^c^The follow-up visits should be scheduled from the day of randomization. A window of ±14 days is permissible from the scheduled date.

^d^According to package insert at weeks 1-4 for sorafenib group patients and after all initial TARE administrations for TARE+sorafenib group patients only.

^e^Sorafenib to be stopped 7 days before subsequent TARE administration in disease progression and restarted 2 weeks after TARE is administered.

^f^^99m^Tc-MAA scan: technetium-99m macroaggregated albumin.

^g^Additional TARE may be administered only after progression if lesions are amenable to treatment.

^h^ECOG: Eastern Cooperative Oncology Group.

^i^Both before and after progression of disease resulting in termination of further treatment.

^j^Hematology tests: white blood cells, hemoglobin, hematocrit, and platelets.

^k^Coagulation tests: prothrombin time, partial thromboplastin time, and international normalized ratio for prothrombin time.

^l^If treatment commences within 14 days of randomization, the clinical laboratory assessments are not required to be repeated.

^m^Prior to progression of disease resulting in termination of further treatment.

^n^MRI: magnetic resonance imaging.

^o^CT: computed tomography.

### TARE+Sorafenib Group

All patients in the study have liver volume measurements and mass calculations with triple-phase CT (Table 1). Eligibility for TARE with TheraSphere microspheres included a pretreatment angiography with administration of technetium-99m macroaggregated albumin (^99m^Tc-MAA) followed by a ^99m^Tc-MAA single-photon emission computed tomography (SPECT) or SPECT/CT scan to determine catheter positioning for treatment, to assess the potential for extrahepatic shunting requiring use of angiographic occlusion techniques, and to determine the lung shunt fraction.

Patients deemed unsuitable for such treatment can proceed to treatment with sorafenib as described above. For eligible patients, TARE is started within 28 days of randomization, prior to the initiation of sorafenib.

Patients with unilobar disease receive a lobar TARE. Patients with bilobar disease receive TARE to the first lobe, and then TARE to the second lobe after at least 28 days after the first treatment but still within 5 to 8 weeks after randomization.

The treatment approach for TARE with TheraSphere microspheres is lobar. Target liver mass and volume are determined by the positioning of the delivery catheter in the hepatic vasculature and the resulting liver area (Couinaud segments) infused. Since there is considerable individual variation in hepatic vascular anatomy, the determination of target liver mass and volume depends on the variant encountered. TheraSphere microspheres are administered at a dose of 120 Gy ±10% to each lobe. If radiation exposure to the lungs exceeds 30 Gy (or 50 Gy cumulative across all planned infusions, estimated during the dose calculation), the TARE dose is reduced to a minimum dose of 90 Gy ±10%. If, after consideration of dose reduction, radiation exposure to the lung continues to be more than 30 Gy (or 50 Gy cumulative), TARE is not administered and sorafenib treatment is initiated. ^99m^Tc-MAA angiography and ^99m^TC-MAA SPECT or SPECT/CT can be repeated after 4 weeks of continuous treatment with sorafenib to reassess lung shunting. If radiation exposure to the lung is less than 30 Gy for a single treatment (or 50 Gy cumulative over all planned infusions) within a target dose of 90 to 120 Gy ±10%, TARE can be administered.

Sorafenib treatment is initiated at least 2 weeks and up to 6 weeks following administration of TARE (Table 1), rather than concurrently, in order to minimize the risk of additive or synergistic adverse events. Sorafenib is administered according to the package insert. Every 8 weeks after randomization, follow-up is conducted as Table 1 shows.

During follow-up, patients in the TARE+sorafenib group who had hepatic progression with lesions amenable to TARE can be retreated under investigator judgment. In that case, sorafenib is discontinued 7 days prior to TARE (equivalent to approximately 5 to 7 half-lives) and resumed 2 weeks after TARE.

For TARE with TheraSphere microspheres, the catheter is placed under image guidance at the same position as for ^99m^Tc-MAA administration. Prophylaxis with a gastric inhibitor (H2 blocker) is recommended.

After the first 20 patients in the TARE+sorafenib group received both TheraSphere microspheres and sorafenib and completed at least 2 weeks of sorafenib therapy, a feasibility safety assessment was conducted. The IDMC reviewed the safety results of both groups in an unblinded fashion. Stopping further enrollment to trial could have been considered if there was either (1) an unanticipated patient death definitely or probably related to the sequential administration of TARE followed by sorafenib, or (2) a pattern of serious toxicity clearly related to the sequential administration of TARE followed by sorafenib as assessed by the IDMC experts based on the severity of disease of the study population.

### Outcome Measures and Definitions

The primary outcome measure of the STOP-HCC study is overall survival time. The secondary outcomes are time to progression, time to untreatable progression, time to symptomatic progression, tumor response, quality of life, and adverse events. Definitions of these outcomes are as follows:

*Overall survival* is the time from the randomization date to death from any cause.

*Time to progression* is the time from randomization to radiological progression according to Response Evaluation Criteria in Solid Tumor (RECIST) v1.1.

*Time to untreatable progression* is the time from randomization to predefined criteria for untreatable progression. For patients randomly assigned to the TARE+sorafenib group, any liver lesion still amenable to TARE is not considered untreatable progression. Untreatable progression is defined as any of the following: intolerance to sorafenib; occurrence of specific contraindications to sorafenib; assessment of progression in the target lesions or occurrence of new lesions after treatment (for patients randomly assigned to the TARE+sorafenib group, a maximum of 2 retreatments with TARE); occurrence of specific contraindications to TheraSphere microspheres or the appearance of lung or intestinal shunts or anatomical constraints not correctable by radiological procedures; confirmed extrahepatic metastases; deterioration of liver function (Child-Pugh score >B7); and clinical progression to ECOG Performance Status score greater than 1. Such deterioration in performance score is observed at 2 subsequent evaluations at 8-week intervals.

*Time to symptomatic progression* is the time from randomization to ECOG Performance Status greater than 1 with or without tumor progression based on RECIST v1.1; deterioration in ECOG status is confirmed at 2 subsequent evaluations at 8-week intervals.

*Tumor response* is categorized according to RECIST v1.1 (change in the sum of diameter of target lesions) based on investigator assessment of baseline versus subsequent follow-up images [34]. The tumor response, according to the modified RECIST and based on a blinded centralized independent imaging assessment, is recorded as an exploratory end point.

*Quality-of-life* assessment is based on the patient-reported Functional Assessment of Cancer Therapy-Hepatobiliary questionnaire. A deterioration in quality of life is a 7-point decline in the total score or death, whichever occurs first.

*The time to deterioration in quality of life* is calculated as the time from randomization to deterioration in quality of life.

The following additional efficacy variables will be assessed.

*Progression-free survival* is defined as the time from randomization until date of radiological progression according to RECIST v1.1 or death due to any cause, whichever occurs first.

*Duration of objective response* will be determined for patients who have a best response of complete response or partial response. Duration of objective response is defined as the time from first date of response of complete response or partial response until date of progression or death due to any cause, whichever occurs first.

*Duration of disease control* will be determined for patients who had a best response of complete response, partial response, or stable disease. Duration of disease control is defined as the time from the first date of response of complete response, partial response, or stable disease until date of progression or death due to any cause, whichever occurs first.

*Depth of response* is defined as the percentage change from baseline to the nadir in the sum of the longest diameters of target lesions.

*Posttreatment tumor shrinkage* is defined as the proportion of patients achieving a 20% or greater decrease in the sum of the longest diameters of target lesions.

Adverse events, serious adverse events, and unanticipated adverse device effects are collected throughout the study and assessed using version 4.0 of the National Cancer Institute Common Terminology for Adverse Events [35].

### Planned Statistical Analysis

#### Study Design and Sample Size

The STOP-HCC study is a phase 3 adaptive trial using a group-sequential design with overall survival as the primary efficacy end point. The study was designed to detect a 3.5-month increase in median overall survival, from 10.7 months in the sorafenib group to 14.2 months in the TARE+sorafenib group (HR 0.754), using a log-rank test. Due to uncertainty in the expected treatment effect, we planned a sample size reestimation, which would allow the sample size to increase in order to detect a 3.0-month increase in median overall survival time, from 10.7 months in the sorafenib group to 13.7 months in the TARE+sorafenib group (HR 0.781).

A maximum of 417 deaths would yield 80% power to detect the target difference in median overall survival (HR 0.754) with a 2-sided alpha of .05 using a group-sequential design with 2 interim analyses. It was estimated that a maximum of 520 patients would need to be recruited over 60 months, with an 18-month additional follow-up period. This includes an adjustment to take account of an assumed 5% of patients who would be lost to follow-up and for whom a date of death was not recorded, and an assumed additional 5% of patients who would be erroneously randomly assigned because they did not meet the eligibility criteria at randomization.

#### Interim and Final Analyses of the Primary End Point

The IDMC will evaluate the overall survival data during 2 interim analyses, which are planned at the observance of approximately, but no less than, 188 and 250 events, with a 2-sided *P* ≤.0151 at either point allowing the study to be stopped early for efficacy. The efficacy stopping boundaries are based on the rho family error spending function with the parameter value ρ=1.5. If the interim analyses do not occur at exactly 188 or 250 deaths, the corresponding efficacy boundaries will be calculated using the rho family spending function with ρ=1.5.

Sample size modification will be considered at the second interim analysis according to a simplification of the promising zone approach described in Mehta and Pocock [36]. The conditional probability boundaries for the decision rules at the second interim analysis are as follows: unfavorable zone (CP2<z): study size to remain at 417 deaths; promising zone (z≤CP2<0.8): study size to increase to 564 deaths; favorable zone (CP2≥0.8): study size to remain at 417 deaths, where CP2 is defined as the conditional probability of rejecting the null hypothesis at the final analysis, given the results at the second interim analysis, and z is a predefined cutoff defined according to the Müller and Shäffer [37] principle to ensure that type I error is controlled.

The final analysis, without a sample size modification, is planned when approximately, but no less than, 417 deaths have occurred. A 2-sided *P* ≤.0363 is required to declare a statistically significant improvement in median overall survival at the final analysis. If the final analysis does not occur at exactly 417 deaths, the corresponding efficacy boundary will be calculated using the rho family spending function with ρ=1.5.

If the sample size increases after the second interim analysis, the final analysis is planned when approximately, but no less than, 564 deaths have occurred, which gives 80% power to detect an improvement in median overall survival from 10.7 to 13.7 months using a log-rank test with a 2-sided *P* ≤.0363 required to declare statistical significance. We estimated that recruitment of approximately 700 patients over 66 months with an 18-month follow-up period would allow for 564 deaths, yielding 80% power to detect a statistically significant improvement in median overall survival with a 2-sided *P* ≤.0363. This includes an adjustment to take account of an assumed 5% of patients lost to follow-up with no recorded date of death, and an assumed additional 5% of patients erroneously randomly assigned because they did not meet the eligibility criteria at randomization.

#### Statistical Analyses

All efficacy end points will be analyzed using a modified intention-to-treat population, defined as patients who met the study eligibility criteria and were randomly assigned. The per-protocol population is defined as the patients in the modified intention-to-treat population without any major protocol deviations that could affect efficacy evaluation; analysis using the per-protocol population will be according to the treatment actually received. The safety population included all randomly assigned patients who received study treatments at least once; analysis will be according to the treatment actually received.

For the primary end point, the Kaplan-Meier method will be used to estimate overall survival curves, and the log-rank test will be used to compare groups. For all secondary end points, comparison between groups will be conducted at a 2-sided alpha of .05, with analyses occurring only at final analysis to determine statistical significance between the groups. For the secondary time-to-event end points (time to progression, time to untreatable progression, time to symptomatic progression, and time to deterioration in quality of life), the Kaplan-Meier method will be used to estimate curves, and comparisons between groups will be conducted using the log-rank test. Tumor response rates will be compared between groups using the continuity-adjusted Newcombe-Wilson test.

#### Poolability and Other Analyses

Univariable Cox regression analyses of the primary efficacy end point, overall survival, and all other time-to-event end points (ie, time to progression, time to untreatable progression, time to symptomatic progression, and time to deterioration in quality of life) will be conducted with the following baseline factors, one at a time, together with randomized group: age group, race, ethnicity, unilobar versus bilobar disease, region, ECOG Performance Status, presence of branch PVTT, duration from date of initial diagnosis of HCC to randomization, tumor burden, presence of extrahepatic lesions, Child-Pugh score, BCLC stage, HCC etiology, prior oncologic treatment for HCC, bilirubin, albumin, albumin-bilirubin score, and alpha-fetoprotein. This will allow an assessment of each of these factors on the study outcomes.

To address the poolability of data across regions, study sites, and sex, multivariable Cox regression analyses of the time-to-event end points will be conducted with the following factors, together with treatment group, and the factors from the univariable analyses that have a 2-sided *P*<.15: region and treatment group by region interaction, enrolling site and treatment group by site interaction, and sex and treatment group by sex interaction, respectively.

Logistic regression analyses of binary end points (ie, objective response rate and disease control rate) will be conducted in the same way as the Cox regression analyses described above.

## Results

Enrollment for the study completed in September 2017. Results of the first and second interim analyses were reviewed by the IDMC; the recommendation of the committee, at both interim analyses, was to continue the study without any changes.

## Discussion

### Overview

TARE has gained a place in various guidelines and consensus recommendations for the treatment of unresectable HCC. A consensus panel report by the Radioembolization Brachytherapy Oncology Consortium states that TARE can be considered for patients with unresectable hepatic primary cancer or metastatic hepatic disease with liver-dominant tumor burden, and a life expectancy of at least 3 months [38]. TARE is recommended in the Asia-Pacific Primary Liver Cancer Expert consensus guidelines in early and intermediate HCC when standard treatment is not compatible and in locally advanced HCC [39]. The Taiwan Liver Cancer Association and the Gastroenterological Society of Taiwan recommend TARE as an option in patients who are refractory to TACE or have either a large tumor burden or major vascular invasion (such as PVTT) [40].

PVTT is the most common form of macrovascular invasion in HCC, and it is a common complication [41]. TACE and other forms of embolic treatment are generally considered contraindicated or not relevant in HCC patients with PVTT. The reason is that multiple TACE treatments could embolize the hepatic artery, leaving the compromised portal vein as the only source of blood supply to the liver and, thus, raise the risk of liver failure [42]. Treatment of HCC associated with PVTT would be a good indication for TheraSphere microspheres because the small size and number of TheraSphere microspheres administered makes them less embolic than other devices, and the ensuing effect on vascular dynamics is smaller than with other devices [43-45]. TARE with TheraSphere microspheres was found to be tolerable and effective in HCC patients with branch or lobar PVTT [44,46,47].

We expect the STOP-HCC study to be the largest study of sorafenib alone versus sorafenib combined with TARE. We chose overall survival as the primary end point; overall survival is the primary end point recommended by the American Association for the Study of Liver Diseases Panel of Experts for the design of clinical trials in HCC patients because it is the most clinically relevant and the one that is least subject to investigator bias [48]. The major factors that may affect overall survival in this trial are the pattern of progression, access to postprogression therapy, tolerance of the combination of TARE and sorafenib, and underlying cirrhosis and hepatitis.

When the study was designed there was no standard second-line treatment available. Now that at least three new drugs are available in second-line treatment (regorafenib, cabozantinib, and the checkpoint inhibitor nivolumab), it is possible that postprogression treatment may affect overall survival. Subsequent treatments that are equally effective in the treatment arms would not be expected to affect the absolute overall survival benefit of the experimental treatment but will make the relative improvement in overall survival smaller, provided that the subsequent therapies used in both treatment arms follow the current standard of care [49]. There is a low risk that postprogression treatment could be a factor influencing the end point. No crossover was permitted in this trial. It is unlikely that TheraSphere, which has a low toxicity profile, will influence the rate of administration of further anticancer treatment at progression or decrease the efficacy of second-line treatment compared with the treatment in the control group [50]. In the recently published REFLECT trial, the median overall survival in sorafenib-treated patients was 12.3 months (95% CI 10.4-13.9) versus 10.7 months in the SHARP trial [11,51]. One explanation could be the proportion of patients receiving treatments after sorafenib (39%) in the REFLECT trial [51].

Another factor that may affect overall survival is the tolerance to the treatment. In the randomized controlled trials of TACE versus TACE plus sorafenib (Sorafenib or Placebo in Combination With Transarterial Chemoembolization for Intermediate-Stage Hepatocellular Carcinoma [SPACE], Sorafenib in Combination With Transarterial Chemoembolisation in Patients With Unresectable Hepatocellular Carcinoma [TACE 2], and Transcatheter Arterial Chemoembolization Therapy in Combination With Sorafenib [TACTICS] trials [52-54]) and of sorafenib versus TARE plus sorafenib (SORAMIC) [32], the combination treatments did not impair TACE or TARE administration. However, SPACE, TACE 2, and SORAMIC did not meet their primary end points. While the TACTICS trial was successful, it could be argued that the favorable results of the combination were mostly related to the low dosage and duration of sorafenib, which was given for a median of 38.7 weeks [55,56] compared with 17.1 weeks (TACE 2), 21 weeks (SPACE), and 29 weeks (SORAMIC). In the TACTICS study, patients received sorafenib 400 mg/day prior to TACE, then 800 mg/day during TACE sessions until time to untreatable progression; the median doses of sorafenib in the TACE 2, SPACE, and SORAMIC trials were 660 mg/day, 566 mg/day, and 485 mg/day [32,52-54], respectively. This low dose in the TACTICS trial allowed for less treatment interruption due to adverse events and a longer duration of treatment. The STOP-HCC study addresses a slightly different population of patients (only advanced, as compared with intermediate stage in TACE 2 and SPACE and 50% at intermediate stage in SORAMIC), and the sensitivity to sorafenib could be different according to the disease stage. Also, to avoid the cumulative side effect of the treatments and allow an effective duration of sorafenib, treatment with sorafenib is started after completion of TARE and dose reduction is planned.

Overall survival should be improved by the control of the different risks of progression. Treatment failure could be the consequence of new intrahepatic lesions, intrahepatic growth, and new extrahepatic lesions (including portal vein thrombosis). TARE should mitigate the first two risks and sorafenib, the third risk.

Finally, survival in HCC patients is confounded by underlying cirrhosis and hepatitis, so while there may be a treatment effect, there are other elements at play that determine survival.

Although the SORAMIC trial did not show an improvement in overall survival, there are some design differences between that trial and the STOP-HCC trial: the STOP-HCC study has a larger sample size and so should have greater statistical power to detect an overall survival difference, the TARE devices used in the 2 studies are different (resin yttrium-90 microspheres vs glass yttrium-90 microspheres), and the SORAMIC trial was conducted in Europe and Turkey with 38 sites, whereas STOP-HCC is being conducted in North America, Europe, and Asia with approximately 100 sites.

We chose secondary end points to determine whether improvement in response affects overall survival and quality of life. The secondary end points of time to progression, time to symptomatic progression, and tumor response rate are also recommended by the American Association for the Study of Liver Diseases Expert Panel [48]. Additionally, this study is assessing time to untreatable progression and quality of life.

### Limitations

Limitations of the study include the unblinded design, but other limitations were minimized as much as possible. Blinding for the sorafenib group would have been difficult to achieve due to its well-known and extensive toxicity profile. There are uncertainties in the assumptions of median overall survival for the sample size calculation (eg, since the population enrolled may include more PVTT patients than expected, and the literature suggests that a larger difference in median overall survival may exist for PVTT patients vs non-PVTT patients), hence the choice of an adaptive study design, which allows the sample size to be increased to detect a smaller difference in median overall survival. The STOP-HCC study addresses this issue in terms of providing a large patient population in a well-designed trial.

### Conclusion

It is important to establish an effective, tolerable, and affordable treatment to improve patient survival for unresectable HCC. One challenge in the treatment of patients with HCC at the advanced stage is providing an efficient treatment of the cancer without impairment of liver function and quality of life. TARE has a limited toxicity profile when used appropriately and, consequently, a low impact on sorafenib dose intensity and duration and is, thus, an attractive concomitant treatment. Enrollment for the STOP-HCC study completed in September 2017 [57], and the estimated follow-up period is 18 months. Data from this trial will enhance the knowledge regarding optimal treatment options for patients with unresectable HCC.

## Appendix

Multimedia Appendix 1 IRB approval of the global principal investigator's site, Northwestern.

## Abbreviations

^99m^Tc-MAA: technetium-99m macroaggregated albumin
BCLC: Barcelona Clinic Liver Cancer
CT: computed tomography
ECOG: Eastern Cooperative Oncology Group
GIDEON: Global Investigation of Therapeutic Decisions in Hepatocellular Carcinoma and of its Treatment With Sorafenib
HCC: hepatocellular carcinoma
HR: hazard ratio
IDMC: independent data monitoring committee
MRI: magnetic resonance imaging
PVTT: portal vein tumor thrombosis
RECIST: Response Evaluation Criteria in Solid Tumors
SHARP: Sorafenib Hepatocellular Carcinoma Assessment Randomized Protocol
SORAMIC: Evaluation of Sorafenib in Combination With Local Micro-Therapy Guided by Primovist Enhanced MRI in Patients With Hepatocellular Carcinoma
SPACE: Sorafenib or Placebo in Combination With Transarterial Chemoembolization for Intermediate-Stage Hepatocellular Carcinoma
SPECT: single-photon emission computed tomography
STOP-HCC: TheraSphere in the Treatment of Patients with Unresectable Hepatocellular Carcinoma
TACE: transarterial chemoembolization
TACE 2: Sorafenib in Combination With Transarterial Chemoembolisation in Patients With Unresectable Hepatocellular Carcinoma
TACTICS: Transcatheter Arterial Chemoembolization Therapy in Combination With Sorafenib
TARE: transarterial radioembolization
