# Supplementary material for: Intra-Arterial TheraSphere Yttrium-90 Glass Microspheres in the Treatment of Patients With Unresectable Hepatocellular Carcinoma: Protocol for the STOP-HCC Phase 3 Randomized Controlled Trial
Source: JMIR Res Protoc. 2018 Aug 15;7(8):e11234. doi: 10.2196/11234 (PMC6115595; doi:10.2196/11234)
Supplement: Multimedia Appendix 1 [file resprot_v7i8e11234_app1.pdf]

Institutional Review Board Office
Northwestern University

Biomedical IRB
750 North Lake Shore Drive
Suite 700
Chicago, Illinois 60611
312-503-9338

Social and Behavioral Sciences IRB
2205 Tech Drive
Hogan Bldg. Suite G-100
Evanston, Illinois 60208
847-467-1723

NORTHWESTERN
UNIVERSITY

2/13/2012

Dr. Laura Kulik
Physician
Medicine

Chicago,IL 60607
lkulik@nmff.org

IRB Project Number: STU00059691
Project Title: DRUG TS-103: A Phase III Clinical Trial of Intra-arterial TheraSphere® in the Treatment of Patients with Unresectable Hepatocellular Carcinoma (HCC)
Project Sites:

Northwestern Medical Faculty Foundation (NMFF)
* Robert H. Lurie Comprehensive Cancer Center
Northwestern University (NU)
Northwestern Memorial Hospital (NMH)

Sponsor Information (Grant #, if applicable):

View Other (Industry Sponsored)

Submission Considered: New Submission **Submission Number:** STU00059691
Study Review Type: Full IRB Review
Meeting Date 2/10/2012
Panel: Panel C
Status: APPROVED **Approval Period:** (2/10/2012 - 2/9/2013)

Dear Dr. Kulik,

The IRB considered and approved your submission referenced above through 2/9/2013 . As Principal Investigator (P.I.), you have ultimate responsibility for the conduct of this study, the ethical performance of the project, and the protection of the rights and welfare of human subjects. You are required to comply with all NU policies and procedures, as well as with all applicable Federal, State and local laws regarding the protection of human subjects in research including, but not limited to the following:

- Not changing the approved protocol or consent form without prior IRB approval (except in an emergency, if necessary, to safeguard the well-being of human subjects).
- Obtaining proper informed consent from human subjects or their legally responsible representative,

using only the currently approved, stamped consent form.
- Promptly reporting unanticipated problems involving risks to subjects or others, or promptly reportable non-compliance in accordance with IRB guidelines.
- Submit a continuing review application 45 days prior to the expiration of IRB approval. If IRB re-approval is not obtained by the end of the approval period indicated above, all research related activities must stop and no new subjects may be enrolled.

IRB approval includes the following:

Written Consent Form/Consent Form and Authorization for Research:
Name
DRUG TS-103 ICF dated 12.05.11.doc

Protocol Document:
Name
DRUG TS-103 Protocol version 1.0 dated 03.09.11.pdf

Recruitment Materials:
Name
DRUG TS103 website abstract

Survey/Questionnaires:
Name
DRUG TS-103 FACT-Hep Questionnaire.pdf

For more information regarding IRB Office submissions and guidelines, please consult http://www.northwestern.edu/research/OPRS/irb.
This Institution has an approved Federalwide Assurance with the Department of Health and Human Services: FWA00001549.
